# Balancing risk, intimacy and (non)compliance: a qualitative study of sex across household during COVID-19 social restrictions

**DOI:** 10.1080/13691058.2022.2078507

**Published:** 2022-06-08

**Authors:** Karen J. Maxwell, Raquel Bosó Pérez, David Reid, Lily Freeman, Dee Menezes, Pam Sonnenberg, Nigel Field, Kirstin R. Mitchell

**Affiliations:** a MRC/CSO Social and Public Health Sciences Unit, University of Glasgow, Glasgow, UK; b Institute of Global Health, UCL, London, UK; c Institute of Health Informatics, UCL, London, UK

**Keywords:** risk behaviour, sexual behaviour, COVID-19, pandemic restrictions

## Abstract

Government controls over intimate relationships, imposed to limit the spread of Sars-CoV-2, were unprecedented in modern times. This study draws on data from qualitative interviews with 18 participants in Natsal-COVID, a quasi-representative web-panel survey of the British population (*n* = 6,654 people), reporting that they had sex with someone from outside their household in the preceding four weeks; a period in which contact between households was restricted in the UK. Whilst only 10% of people reported sexual contact outside their household, among single people and those in non-cohabiting relationships, rates were much higher (Natsal-COVID). Our findings show that individuals did not take decisions to meet up with sexual partners lightly. Participants were motivated by needs—for connection, security, intimacy and a sense of normality. People balanced risks—of catching COVID-19, social judgement and punishment for rule-breaking—against other perceived risks, including to their mental health or relationships. We used situated rationality and social action theories of risk to demonstrate that people weighed up risk in socially situated ways and exhibited complex decision-making when deciding not to comply with restrictions. Understanding motivations for non-compliance is crucial to informing future public health messaging which accounts for the needs and circumstances of all population members.

## Introduction

Following the declaration of COVID-19 as a global pandemic by the World Health Organisation in March 2020, governments around the world sought to slow virus transmission through stringent measures including lockdowns, physical distancing and reducing contact with others (Lewnard and Lo 2020; Buchholz 2020). By late Spring 2020, over one third of the world’s population was in lockdown (Buchholz 2020), effectively restricting the movement and social interactions of billions of people. In the UK, a national lockdown was announced on 23^rd^ March 2020, with the government issuing a ‘stay at home’ order to all citizens and prohibiting all non-essential contact between households (Institute for Government 2021). This lockdown remained in place for around three months, with restrictions gradually easing from mid-June 2020. After this point, individuals from separate households were once again allowed to meet under limited circumstances (outdoors and with 2 metres distancing).

Research into the effects of pandemic restrictions on sexual behaviour suggested far-reaching changes varying by population sub-group (Hille et al. 2021; Lehmiller et al. 2021). Studies reported decreases in sexual frequency (Hille et al. 2021; Lehmiller et al. 2021), decreases in sexual satisfaction (Cocci et al. 2020), changes in sexual desire (Balzarini et al. 2021; Cocci et al. 2020; Lehmiller et al. 2021) and increases in use of digital practices such as cybersex and sexting (Döring 2020). For single people, many changes were linked to compliance with government-mandated physical distancing guidelines, which made engaging in any kind of partnered sexual activity more difficult and socially stigmatised (Döring 2020; Rodrigues et al. 2021; Gilbert et al. 2021). As might be expected, single people reported fewer sex partners and less risky sexual behaviour (Lehmiller et al. 2021; Shilo and Mor 2020; Rodrigues et al. 2021). However, those reporting more sexual partners despite restrictions reported feelings of stigma (Gilbert et al. 2021). For those in relationships, changes in sexual frequency, desire and satisfaction were reported, likely linked to COVID-related stressors and greater partner conflict during the pandemic (Luetke et al. 2020; Mitchell et al. 2022).

Less attention has been given to understanding how single people seeking new partners or those in non-cohabiting relationships navigated social restrictions. The impact of restrictions on those living alone is particularly important to understand, given increasing concern over the impact of the pandemic on mental health and loneliness (Groarke et al. 2020; Killgore et al. 2020). The ‘choice’ for these individuals was typically between abstinence or rule breaking. In the making of rules, it was apparent that their circumstances were side-lined by a dominant view of ‘households’ as family units and married/cohabiting couples (Williams 2021; Döring 2020; Rothmüller 2021), despite the fact that in the UK 40% of people do not live in a couple, rising to 71% amongst 16 to 29-year-olds (ONS. 2020). As Williams wrote in the Guardian newspaper ‘arguably, many of us are only rule-abiding because the rules acknowledge us’ (Williams 2021). In mid-June 2020, the UK government introduced legislation allowing individuals living alone to form a ‘support bubble’ with another household meaning that individuals in joined households could meet indoors and, crucially, stay overnight. Prior to this, several high-profile public figures were ‘caught’ breaking covid rules, including for intimacy reasons (BBC News 2020), with consequent potential to undermine public adherence. This paper provides a qualitative account of the motivations and decision-making of people who did not comply with social mixing restrictions for intimacy reasons, shedding light on the experience of social restrictions for those least well accommodated by the restrictions.

### Theoretical framework

In seeking to understand individuals’ risk behaviour during the pandemic, we draw on situated rationality and social action theories of risk. *Situated rationality theories* are concerned with how individuals make decisions about risk (Rhodes 1997; Pound and Campbell 2015). These theories propose that risks and their perception are context-dependent and that risk-taking should be seen as *socially-situated* (Bloor 1995). Individuals make decisions about risk in the context of other risks which may be considered more immediate. *Social action theories* meanwhile view risk behaviour as the product of social interactions (Rhodes 1997). Risk behaviour most often is not simply an individual ‘action’ but instead an outcome of negotiated actions within social relationships. People make decisions about risk and what is considered risky in the context of their relationships, peer group norms and wider cultural norms. Thus, in the case of sexual behaviour, a ‘decision’ to meet up with a sexual partner during a pandemic is not simply an individual ‘decision’ but rather a negotiated action influenced by the views, attitudes and risk-perceptions of partners and wider social networks.

These risk perspectives originate in the study of gay men and injection drug-users’ risk decision-making in relation to the HIV pandemic in the 1980s. It is noteworthy that such perspectives have helped to understand how behaviour that may to outsiders seem reckless or risky can be interpreted as rational when viewed through the lens of situated rationality. Both the HIV pandemic of the 1980s and the current COVID-19 pandemic call for new understandings of how people make complex sexual health risk decisions under conditions of enormous stigma, moral judgment and scientific uncertainty. In the context of the current study, such theoretical perspectives can help understand behaviour that might be otherwise seen as risky, deviant and irresponsible in the context of the rapidly unfolding COVID-19 pandemic.

### Our study

Natsal-COVID is a quasi-representative population-based study, which used mixed methods to investigate sexual attitudes, practices, and access to sexual and reproductive health (SRH) services in Britain during the COVID-19 pandemic. This paper draws on qualitative interviews with participants reporting sexual contact with a partner outside their household during a time of restricted social contact. Four months into the UK pandemic, around 10% of the general population reported intimate contact outside their household, but amongst those in non-cohabiting relationships, this was 56%, and for single people, 37% (Sonnenberg et al. 2022). The current paper qualitatively explores the motivations and decision-making of people reporting sexual contact against regulations, to better understand adherence with pandemic guidelines.

## Methods

### Study design and participants

The Natsal-COVID study is a mixed methods study aiming to understand how the COVID-19 pandemic affected sexual lives and sexual health in Britain. Wave 1 consisted of a quantitative web-panel survey of 6,654 people, using quota-based sampling and weighting to achieve a quasi-representative sample of the British general population, and a qualitative follow-on study, purposively sampling participants to investigate specific topics of interest. Further details of the survey design and methods, including sampling and participant characteristics have been previously reported (Dema et al. 2021). The survey included a set of questions about the type and circumstance of any sexual experiences in the four weeks prior to survey, including whether this was with a person living in the same or a different household (full survey available at https://www.natsal.ac.uk/natsal-covid-study). The Wave 1 survey was conducted in July to August 2020, with follow-up interviews completed in October and November 2020. At the time of survey, some social mixing restrictions had been eased, and ‘social bubbles’ legislation had been introduced, but at no stage was meeting with a partner from another household allowed (see timeline in Sonnenberg et al. 2022).

Participants in the qualitative follow-on study reported of one (or more) of the following to the survey and agreed to follow-up: (1) intimate contact with someone outside their household; (2) unmet need for SRH; or (3) relationship difficulties. This paper focuses on data from participants reporting intimate contact with someone outside their household. In total, *n* = 741 reported intimate contact with someone outside their household. Of these, *n* = 468 (63%) agreed to follow-up. We quota sampled from this group (*n* = 468) to ensure a sample with variation by age, ethnicity, gender and region. Participant characteristics of interviewees can be seen in Table 1.

**Table 1.** Participant characteristics (number**)**.

**Gender**	Man (9)
	Woman (9)
**Age (years)**	18–29 (5)
	30–39 (5)
	40–49 (4)
	50–59 (4)
**Ethnicity**	Asian British, Asian (2)
	Black British (1)
	White British (15)
**Sexual orientation**	Gay (2)
	Heterosexual (16)
**Region**	London (4)
	Midlands (1)
	North-East (2)
	North-West (3)
	South-East (2)
	South-West (2)
	Scotland (3)
	Wales (1)
**Relationship Status**	In a relationship (10)
	Single (8)

Ethical approval for the study reported here was obtained from University of Glasgow MVLS College Ethics Committee (20019174) and London School of Hygiene and Tropical Medicine Research Ethics committee (22565).

### Data collection

Individuals were contacted by the research team to confirm eligibility and provide further information about the study. Those who indicated that they were interested were emailed the study information sheet and given time to decide if they would like to take part. Consent forms were completed, signed, and emailed back to the study team prior to interview. Due to the ongoing COVID-19 pandemic and imperative to reduce face-to-face contact, interviews were conducted via telephone (*n* = 17) or videocall (*n* = 1), as per participant preference. Consent was also recorded verbally at the start of each interview, separate to the interview recording. Eighteen interviews were conducted between October and November 2020 by three trained qualitative interviewers (DR, KJM and RBP) and lasted on average 1 h (range: 45 min to 1 h 20 min). Interviews focused on circumstances surrounding the sexual contact with partner(s) outside of their household, motivations for contact, perceptions of risk (sexual, COVID-19, social), and measures taken to mitigate risks. Fieldnotes were taken after each interview, to add context and summarise interviewer reflections on the interview. Participants were given a £30 e-voucher to thank them for their time and contribution.

### Data analysis

Interview data were transcribed verbatim by a professional transcription agency and the transcripts entered into NVivo 12 to facilitate data analysis. Data were analysed using a structured form of thematic analysis, framework analysis (Ritchie and Spencer 2002). In framework analysis, a matrix is produced with cases (participants) as rows and themes as columns, allowing for a systematic comparison of themes across all participants. In summarising participants’ views in a matrix, participants’ views on each theme remain connected to other aspects of their account so that the context of the individual’s views is retained. In common with other types of thematic analysis, the focus remains on identifying commonalities and differences in the data in relation to generated themes.

Coding and analysis were undertaken by KJM, RBP, DR and KRM. A coding framework was designed to explore participants’ circumstances and motivations surrounding their sexual contact during restrictions. This was developed and refined using both inductive and deductive coding. For example, we began inductively by using the initial coding framework to analyse three transcripts and noting themes arising in these transcripts around participant understandings of their motivations and circumstances. Discussion between analysts of these initial themes led to consideration of theoretical frameworks most apposite to understanding and interpretation of the phenomena. A decision to focus on risk-theory then informed deductive generation of further codes, designed to reflect our analytical approach. All transcripts were coded to this refined coding framework (see Appendix 1). Emerging themes and their interpretation were discussed at regular meetings between analysts.

## Findings

Participants’ accounts made clear that decisions to meet up with a sexual partner at a time of restricted social contact were motivated by needs, and that risks were consciously considered in decision-making. In particular, participants considered risk in socially-situated ways. Thus, our findings are discussed under two broad, over-arching headings: motivations and socially-situated decision-making.

### Motivations

#### Need for connection

For both single people and those in relationships, there was evidence of a need for human connection. Fourteen of eighteen participants spoke openly about or alluded to feeling a need for connection, showing how fundamental this need is to human experience. Laura, a married heterosexual woman who lived apart from her partner, spoke of her overwhelming need to see him physically, despite awareness of COVID-19 transmission risks and personal experience of loss to COVID-19. Susan also described the difficulty of not being able to see her partner in person during the early stages of lockdown:

Just having someone’s arms around you and, you know, holding hands, and all this sort of thing, all the things that you take for granted, that you think, “oh God. You just miss that human interaction with that one person”, don’t you? (Susan, female, 50–59, heterosexual, in a relationship)

Connection was not simply about physical intimacy; it was about a physical sharing of everyday life. Neil and Aaron, both in heterosexual non-cohabiting relationships, discussed craving the physical presence of their partner and simply doing ‘normal’ things together, such as watching TV or sitting on the sofa together. Those in non-cohabiting relationships found it hard to be kept physically apart from their partners, with an additional complication being uncertainty about how long restrictions would last.

For single people, the need for human connection was felt starkly, with participants referring to loneliness and a lack of physical contact increasing their feelings of anxiety and insecurity. For Liam, who was living with his sister and her family, there was a sense of panic about the loss of physical contact:

I was just panicking about ever having human contact again. I know that seems a bit silly because I was just like what if the world suddenly becomes a world where we never ever get to shake hands or hug anyone ever again. (Liam, male, 18–29, gay, single)

And for Hope, an overseas student who spent the majority of lockdown alone in her room in student accommodation, the lack of physical contact exacerbated her feelings of anxiety:

It was definitely a very anxiety-provoking, stressful time, I think there was a constant sense of need; the sense of need, sort of, arose a lot more in terms of emotional investment you would need, the physical investment you would need. Just that sense of feeling safe and secure, like a hug could provide, for example, where you couldn’t really hug your family, you couldn’t really hug anybody for that matter (Hope, female, 18–29, heterosexual, single)

#### Need for sexual intimacy

Sexual intimacy needs were more commonly expressed by single participants; those in relationships tended to downplay sexual intimacy as a need. However, this was not exclusively the case, with both Janey and David, in heterosexual non-cohabiting relationships, making reference to their sexual needs in their discussions of their motivation to see their partner.

Single participants spoke of craving physical touch, and related this to other needs, for example ‘human contact’ or feelings of security. For these participants, physical touch and sex were described as bringing comfort, closeness, and security during a time of uncertainty. For example, Hope’s craving of physical touch above and Liam’s description of his desire for ‘human contact’:

I think it was just a really desperate attempt to get human contact because the idea of having another man’s touch was something that I was desiring. I felt like when there were passionate moments it felt like I was able to be close to someone again and I don’t know if I found comfort in that somehow, that I wanted a bit more of it whenever I felt really down. (Liam, male, 18–29, gay, single)

#### Need for normality

A common motivation for meeting up was to maintain or seek a feeling of ‘normality’ in an otherwise abnormal set of circumstances. For those in relationships, this manifested as a desire to continue as before, making little change to everyday routines. In these cases, meeting up with a partner was less a consciously made decision and more a continuation of everyday life, despite restrictions. These participants tended not to offer explicit explanations of their motivations, commenting ‘we just carried on as before’ (Janey, heterosexual, 50–59) or of how they tried to ‘keep as normal as possible’ (Lee, heterosexual, 40–49).

For others, notably more often those who were single, the need for normality was expressed more as a longing for a brief hiatus from the mental load of anxiety brought about by the pandemic, or of seeking to exercise agency in a context of little control. For example, Liam described continuing to seek and meet up with sexual partners through apps during the social contact restrictions stating he ‘just wanted it to feel normal’.

#### Need for security

For a minority, reaching out for sexual contact appeared to be motivated by a need for security. Katherine, Liam and Hope, all single at the start of the pandemic, experienced long periods of time alone each day, which provoked feelings of loneliness and anxiety. Against this backdrop, they explained seeking sexual contact as a way of seeking security in uncertain circumstances. As Hope explained:

For both of us [her and male friend], we don’t really have a lot of contact and there is this, you know, this constant sense of loneliness that’s there. So I think, you know, it was just a lot of […], physical and emotional lack of… the same need that’s there for you to feel safe and secure, and it [the sex] did definitely provide that at that point in time. (Hope, female, 18–29, heterosexual, single)

Although only a minority explicitly expressed that they were seeking security in reaching out for sexual contact, many more participants implied this in their discussions. Participants described how restrictions had removed sources of security from their lives by taking away their everyday interactions with partners. For example, Lee, a recently divorced heterosexual man in a new relationship, described how the inability to physically be with his partner had caused him to feel insecure about his fledgling relationship.

### Socially-situated decision-making

#### Weighing up risk

People made decisions about whether to meet up with a sexual partner in the context of other perceived risks, such as risk to their mental health or relationship stability. For some, the physical health risks of COVID-19 were perceived to be high due to their or their partner’s underlying health conditions. However, for most, the perceived risk to mental health was held as more significant than the perceived risk of COVID-19 to their physical health:

Where obviously I couldn’t see her during lockdown, she was getting extraordinarily depressed herself because she suffers with depression naturally anyway and all she was basically doing was [working], had no release, stuck at home on her own. […] And we reached a point […] we basically said, ‘Sod it, I’ll come over to you’, so that’s what we did. […] Totally illegal I appreciate that but I think it’s healthy for both of us to do so. (David, male, 50–59, heterosexual, in a relationship)

The level of risk of catching COVID-19 through meeting one’s partner was also contrasted against the perceived risk of catching COVID-19 during other behaviours, such as going into work, taking public transport, or going to the supermarket.

To be honest when you say you’re going to see somebody from a different household, I’m going to see somebody I love, I don’t give a toss what you say. I’m not spreading anything. […] I’m washing my hands. Doing everything you’ve told me to do. […] It’s no different than me going to [supermarket] (Aaron, male, 30–39, heterosexual, in a relationship)

In making their decisions, participants thought about their perceived level of risk of catching COVID-19 through meeting up with their partner. Meeting was more likely where risks were perceived to be low:

So, we weighed up the risk of me catching it and passing it to him was very, very slim so we decided that was a risk we were OK with taking. (Pamela, female, 30–39, heterosexual, in a relationship)

For participants like Aaron who ‘saw minimal risk’, the risk of COVID-19 was perceived to be low because both partners were living alone and seeing very few (or no) other people. Participants also described taking mitigating actions in other parts of their lives, ‘being extra careful everywhere else’, in order to be able to continue seeing their romantic partner. For example, Janey rationalised her continued contact with her non-resident partner due to their low contact with anyone but each other:

I was being, you know, I’m not…I’m not seeing anybody else, so I’m not really putting anybody else at risk is what I thought. […] I wasn’t in contact in any, you know, way, shape or form with my family, so I figured, we’re not really doing anybody any harm. (Janey, female, 50–59, heterosexual, in a relationship)

Participants described making changes elsewhere in their lives (not seeing friends or family, not travelling on public transport) to enable them to continue seeing their romantic partners. The decision to meet up with one’s partner was often characterised as ‘a risk worth taking’:

It was a risk I was willing to take, and clearly he was. You know? For the benefit of my mental health, I suppose you would say, it was worth it to me, you know? (Laura, female, 40–49, heterosexual, in a relationship)

Thus, participants were aware of risk and of their behaviour being potentially ‘risky’, but risks were seen as low and thus, outweighed by the potential benefits of seeing one’s partner. The exception to this was two single participants who met up with sexual partners despite assessing the risk level as relatively high. In both cases, risk was weighed against need (in this case sexual need) and the need felt to be greater. Both Jack (a single straight man) and Liam (a single gay man) described meeting new partners through dating apps during lockdown and described conflicted feelings of both justification and guilt throughout their interviews.

I don’t think in that moment that [risk of catching COVID-19] was even going through my head. I mean the fact I was already meeting them; I might have already lost all my common sense […] After I finished meeting them, I would eventually be like ‘what am I doing meeting with people, doing this?’. I do feel guilty. I still feel guilty that in April I was a bit irresponsible still meeting for, you know, for that [sex], when I shouldn’t have been. (Liam, male, 18–29, gay, single)

#### Social influences on decision-making

Participant accounts reflected how decisions to meet up with a sexual partner were not taken individually, but influenced by partners, peers, and other social contacts (such as neighbours). There was evidence of conversations taking place between partners, of partners’ wishes being taken into consideration, and of mutual decisions being taken. Partners’ views were typically pivotal. Partners’ perceptions of risk sometimes differed, and here the wishes of the more risk-averse partner often took precedence. For example, Lee, described how he complied with his ‘really strict’ partner in her wish not to meet until they both felt that the risk level was low enough.

A sense of decision-making being a result of negotiated actions involving more than one actor is evident in Hope’s description of how she and a male friend made the decision to have sex, following open discussion of how they were coping and their needs:

There was a conversation and there was this, you know, this constant sense of loneliness and […] a sense of companionship that’s lacking having stayed in your room all the time. […] both of us were very open about it [having sex] and I was just sharing how I’m coping, how he’s coping […] And it just…we just decided that, you know what, let’s just… because both of us, sort of, feel that it’d make us feel better, and that’s something that we think would help us in terms of anxiety and in terms of how we feel. (Hope, female, 18–29, heterosexual, single)

Among single participants, perceived peer group norms were influential. For Jack, a single heterosexual man in his 30s who used dating apps to meet new partners during lockdown, decision-making was influenced by his sense that ‘everyone else was still dating.’ Jack described a peer-group in which his many single friends in their 30s continued using dating apps throughout the period of lockdown, thus justifying his own behaviour.

Liam, a gay man in his twenties also expressed his sense of relief on talking to a close friend and discovering that others had also met up with people during lockdown. This feeling of being legitimised by others in his peer group lessened his feelings of guilt and provided reassurance that he was not the only one:

I just talked about how sometimes I did feel guilty and he was like “No need to feel guilty”. They were just convincing me that everyone needs a bit of release. […] I know some friends, like me, have done the same, they have just had to meet with people. (Liam, male, 18–29, gay, single)

In contrast, there was also evidence of peer group norms working in the opposite direction; as a reminder to adhere to social mixing restrictions. Several participants spoke about being careful who they revealed their contact with romantic or sexual partners to for fear of social judgement.

I felt I had to be careful because […] I was doing something that was perhaps against the rules at the time. So, I was careful about what I said to whom. (Katherine, female, 40–49, heterosexual, single)

Participants were also aware of the potential for social judgement from neighbours, as people who were perceived as ‘watching’ the comings and goings at one’s home and potentially disapproving of participants’ actions. Although these fears of social censure did not stop participants from meeting up with partners, they were aware of the threat of social judgement.

I will say that she [partner] was more worried about the judgement, yeah, not just from her housemate, it was more from the neighbours (Jack, male, 30–39, heterosexual, single)

#### Broader social context influencing decision-making

Decisions about meeting up with sexual partners were made against a backdrop of unprecedented state regulation of private lives and legal sanctions for non-compliance. Most participants were more fearful of social judgement than of legal repercussions, but there were clear indications that some participants had thought about the legal ramifications. David, a retired police-officer, described how he was careful to take a bag of shopping every time he visited his partner in case he was stopped by the police. Other participants worried about people reporting them. Often, they were as concerned about the shame of being caught transgressing than about the punishment per se.

I think that it was just concern in case—even in case somebody has seen us, and someone decided they would like to phone kind of like, tell some authorities. (Billy, male, 18–29, heterosexual, in a relationship)

The wider social context also encompassed an awareness of increased regulation of social and intimate relationships. Participants’ accounts demonstrated resistance to this increased regulation of intimate lives. In many accounts, there was evidence of a strong desire to be able to make one’s own decisions regarding one’s romantic or sexual relationships; a belief that one could make the best judgement of risk for oneself. This was more evident for those in relationships. These participants felt strongly that they should be able to make decisions about their intimate lives for themselves, and resented government regulation of their sexual relationships.

I think we waited that and when they come back with some other ridiculous decision we thought, “Oh, we’re not– I’m not standing for this anymore it’s nonsense”. […] I just feel, I feel quite a strong sense of resentment that we were put in this position when we didn’t need to be. […] I just—why were we kept apart? (David, male, 50–59, heterosexual, in a relationship)

Several participants expressed feelings of indignation at their romantic relationship becoming subject to government regulation. Resistance to state controls on intimate relationships was often strongly underpinned by a feeling that such controls were not justified. Those who did continue seeing their partner throughout the strict lockdown often questioned the rationale behind the rules and felt that they were better placed to make considered risk assessments for their own personal circumstances than the government.

So, your rule that I’m not allowed to go and see somebody from a different house is bullshit.

Interviewer: Because?

Because of risk. There’s no risk because I’ve got bloody alcohol wipes in my pocket. I’ve been nowhere today. I’ve been stuck in the house. I’ve walked the two miles to go and see my missus, and I’m sorry, that’s not, I’m not a risk to anybody. (Aaron, male, 30–39, heterosexual, in a relationship)

## Discussion

Study findings offer a new perspective on how individuals assess and weigh up risks in sexual decision-making at a time of social restriction. Arguably, in Britain, as in many other countries, pandemic restrictions minimised the needs of those who were not in heteronormative, co-habiting couple relationships, leaving many feeling unrecognised by new social regulations (Williams 2021; Rothmüller 2021). This includes our sample, who were single and seeking new relationships or in non-cohabiting relationships at the time of pandemic restrictions, and thus for whom social restrictions demanded greater personal sacrifice. Our findings indicate that, for these people, experiencing long periods with no physical contact was challenging and provoked feelings of loneliness and anxiety. Thus, non-compliance was motivated by a weighing of risk that minimised physical risks to health and prioritised benefits to mental health and relationship security of seeing one’s sexual partner(s).

Study findings suggest that individuals who met up with a sexual partner during a time of social mixing restrictions weighed up risk in socially situated ways. In other words, some risks were given greater weight than others depending on an individual’s priorities and the social context. Participants weighed up risks—of catching COVID-19, of social judgement, of potential punishment - against other risks such as the detrimental effect on their relationship or mental health. For many, the risk to their mental health from separation and isolation weighed more heavily in decision-making than the perceived risks to physical health of meeting up. Our data show how a behaviour that might seem risky, reckless and selfish to others, was rational to these individuals, given their assessment of the context and perceived risks. This is consistent with situated rationality theory; the idea that some risk-taking is perceived as rational by the risk-taker as certain risks are deemed more salient than others depending on the social context.

The exception to this in our data could be found in two single participants who *did* recognise their behaviour as high risk and subsequently felt guilty. Interestingly, these participants were able to rationalise their behaviour through comparison with others in their peer groups who they felt were behaving similarly, consistent with social action theories of risk which ‘shift the unit of analysis from the individual to the social relationship’ (Rhodes 1997, 216) and highlight how risk decisions are the product of ‘negotiated actions’ made within social contexts. Our data illustrate how decisions were socially influenced; those who were single were influenced by what they perceived others were doing or accepting, while those in relationships negotiated the decision with their partner.

Whilst this study is able to shed light on motivations and weighing of risk in sexual decision-making during time of unprecedented government regulation of social relationships, we acknowledge the limited number of sexual and gender minority participants in this study, particularly trans and non-binary people, limiting our ability to comment on specific challenges and experiences for these populations. In particular, the majority heterosexual accounts presented here may represent heterosexual privilege in that for the majority of participants, this was the first time they may have experienced surveillance of their sexual behaviour or sexual stigma in ways that have been commonplace for other minority groups for decades. Work specifically with men who have sex with men concludes that COVID-19 control measures were likely to amplify health inequalities through creating further stigma for gay men (Gaspar et al. 2022). Further research into the particular challenges for sexual and gender minority people during this period would be helpful.

In the UK, as elsewhere, pandemic-related social regulations focused heavily on ‘households’, effectively legitimising some kinds of relationships (co-habiting relationships, nuclear families) and delegitimising others (non-cohabiting relationships, casual sexual relationships, non-monogamous relationships) (Rothmüller 2021). Thus, for many people, including our study participants, rules may have seemed to unfairly prioritise the needs of some citizens over others (Long et al. 2022). In the making of rules, arguably psychological and sexual needs were largely ignored or assumed to be equivalent across all population groups, effectively disadvantaging some demographic sub-groups more than others. Research suggests that sexual minorities experienced more exclusion than heterosexuals during pandemic restrictions, being more isolated from their communities (Gaspar et al. 2022; Rothmüller and Rack 2021). Similarly, young people may have been particularly vulnerable to social isolation, being less likely to live with romantic partners (Birditt et al. 2021). Individuals who felt disadvantaged by the rules were more likely to break them, and to resist or resent authority (Clotworthy and Westendorp 2020; Rothmüller 2021). In the context of our study, this was evident in participants’ reports of disillusionment with the rules and resistance to the idea that governments could dictate their sexual choices.

Williams (2021) argues that it is ‘not beyond the wit’ of modern societies to accommodate complexities of modern relationships in the making of rules, and points to the Belgian inclusion of *Knuffelcontacts* (‘hug buddies’) within social restrictions, which effectively allowed people to have intimate contact during the pandemic. In the UK, new expectations that individuals stay physically distanced from their romantic/sexual partners for long periods, combined with the breaking of rules by several high-profile figures, proved to be too much for many. This paper has shed light on the motivations of those who did not comply with COVID-19 restrictions. Understanding how intimacy needs conflict with adherence to pandemic restrictions is important as this can aid our understanding of how to better design public health messaging in the future. In particular, rules and guidance must take into account the needs and circumstances of all members of the population, and not only those who fit into a narrow set of normative criteria (e.g. married or cohabiting couples and families).

### Strengths and limitations

This study provides new insight into sexual decision-making during the COVID-19 pandemic. Its location within a larger population-quasi-representative study (Natsal-COVID) enabled a strong sampling methodology, allowing for quota sampling from different population groups, ensuring representation of a wide range of views. We captured views from younger and older adults, men and women, gay and heterosexual participants, from all over Britain. However, as with all qualitative research, this study draws on a small sample and it is the analytical themes, rather than the data itself that is intended to be generalisable.

Despite attempts to recruit a broad sample, our sample included a relatively small number of sexual and gender minority participants and no participants identifying as non-binary or trans. This is a limitation as studies point to these populations experiencing extra burden and stigma during the pandemic, perhaps leading to even stronger motivations for non-compliance with restrictions (Rothmüller 2021). Furthermore, our sample did not include anyone in a non-monogamous or polyamorous relationship. Inclusion of these groups would shed further light on the experiences and challenges of these under-represented groups at this time.

One potential limitation of this research is our exclusive focus on non-adherence. Because we did not interview people in non-cohabiting relationships who *did* adhere to social mixing restrictions, we are unable to address the question of why some people did comply with restrictions whilst others did not. Further research into the risk perceptions of those who did comply and their reasoning would be useful.

As with any self-report research, there is potential for social desirability bias. Participants were asked to report on sensitive behaviours at a time when these behaviours went against social taboos. Although every effort was made to assure participants of confidentiality, there may have been things that people would have liked to say that they felt they could not. Thus, our findings, whilst providing evidence that policy makers should take people’s needs for physical contact with romantic/sexual partners seriously, may actually understate the extent of the problem. Our findings also reflect participants’ retrospective accounts of their behaviour, meaning we cannot be certain whether these accounts reflect their feelings at the time or their post-hoc rationalisation of behaviours, having had time to reflect on them.

### Conclusion

Findings from this study add to a small but growing body of knowledge around compliance with pandemic social restrictions and sexual behaviour during the pandemic. The study provides insight into people’s risk decision-making, motivations and needs during a time of unprecedented social restrictions. Our findings demonstrate that people who reported meeting up with a sexual partner at a time when this was prohibited weighed up risks in socially situated ways, with some risks taking precedence over others. Decisions about meeting up with sexual partners were not taken lightly, but physical contact was deemed important and thus rationalised.

Social restrictions did not place equal burden on everyone; some population groups were disproportionately affected by restrictions. Our findings suggest that, amongst those who did not adhere to restrictions, there was a strong sense of the unfairness of the rules and of the toll social restrictions had taken on mental health and relationship stability. Whilst we acknowledge that lockdowns were extremely effective at controlling COVID-19 transmission, we argue that governments and policy makers need to pay heed to psychological and intimacy needs and acknowledge these in future restrictions.

Understanding how sexual needs conflict with adherence to pandemic restrictions is important to understanding public adherence more generally and to communication of public health messages in the future. Given the ongoing nature of the COVID-19 pandemic, it is important that the differential impacts of proposed intervention strategies are considered and acknowledged in the communication of messaging and other public health responses.

## Appendix 1. Coding framework

**Table:**

1. Sexual rights
Inequalities
Reference to government guidance
2. Risk
Balancing risks/assessing risk
Reputational risks
Risk of COVID
Rules NOT risks
Sexual risks
3. Needs
Autonomy
Competence
Relatedness
Intimacy needs
Mental Health Needs
Boredom
Practical needs
Security needs
4. Other
Attitudes towards COVID
Changes in dating practices
Decision making
Guilt
Nothing changed
